# Microsatellite loci for *Urochloa decumbens* (Stapf) R.D. Webster and cross-amplification in other *Urochloa* species

**DOI:** 10.1186/s13104-016-1967-9

**Published:** 2016-03-10

**Authors:** Rebecca C. U. Ferreira, Letícia J. Cançado, Cacilda B. do Valle, Lucimara Chiari, Anete P. de Souza

**Affiliations:** Centro de Biologia Molecular e Engenharia Genética (CBMEG), Universidade Estadual de Campinas (UNICAMP), Cidade Universitária Zeferino Vaz, CP 6010, Campinas, SP CEP 13083-875 Brazil; EMBRAPA Genetic Resources and Biotechnology, Brazilian Agricultural Research Corporation, CP 02372, Brasília, DF CEP 70770-900 Brazil; EMBRAPA Beef Cattle, Brazilian Agricultural Research Corporation, CP 154, Campo Grande, MS CEP 79002-970 Brazil; Departamento de Biologia Vegetal, Instituto de Biologia, Universidade Estadual de Campinas (UNICAMP), Cidade Universitária Zeferino Vaz, CP 6109, Campinas, SP CEP 13083-862 Brazil

**Keywords:** Enriched library, Forage, Signalgrass, Simple sequence repeat, Transferability

## Abstract

**Background:**

Forage grasses of the African genus *Urochloa* (syn. *Brachiaria*) are the basis of Brazilian beef production, and there is a strong demand for high quality, productive and adapted forage plants. Among the approximately 100 species of the genus *Urochloa*, *Urochloa decumbens* is one of the most important tropical forage grasses used for pastures due to several of its agronomic attributes. However, the level of understanding of these attributes and the tools with which to control them at the genetic level are limited, mainly due to the apomixis and ploidy level of this species. In this context, the present study aimed to identify and characterize molecular microsatellite markers of *U. decumbens* and to evaluate their cross-amplification in other *Urochloa* species.

**Findings:**

Microsatellite loci were isolated from a previously constructed enriched library from one *U. decumbens* genotype. Specific primers were designed for one hundred thirteen loci, and ninety-three primer pairs successfully amplified microsatellite regions, yielding an average of 4.93 alleles per locus. The polymorphism information content (PIC) values of these loci ranged from 0.26 to 0.85 (average 0.68), and the associated discriminating power (DP) values ranged from 0.22 to 0.97 (average 0.77). Cross-amplification studies demonstrated the potential transferability of these microsatellites to four other *Urochloa* species. Structure analysis revealed the existence of three distinct groups, providing evidence in the allelic pool that *U. decumbens* is closely related to *Urochloa ruziziensis* and *Urochloa brizantha*. The genetic distance values determined using Jaccard’s coefficient ranged from 0.06 to 0.76.

**Conclusions:**

The microsatellite markers identified in this study are the first set of molecular markers for *U. decumbens* species. Their availability will facilitate understanding the genetics of this and other *Urochloa* species and breeding them, and will be useful for germplasm characterization, linkage mapping and marker-assisted selection.

**Electronic supplementary material:**

The online version of this article (doi:10.1186/s13104-016-1967-9) contains supplementary material, which is available to authorized users.

## Background

It has been estimated that 167 million hectares of pasture land in Brazil is used to feed a herd of approximately 208 million head of cattle [1]. These pastures consist mainly of forage grasses of the genus *Urochloa* (syn. *Brachiaria*), which were introduced from Africa [2]. These forage grasses have greatly contributed to the development of the national cattle industry of Brazil, establishing Brazil as the second largest beef producer and the main beef exporter in the world. The competitive advantage of cattle production in Brazil is the exclusive use of pasture [3]. Moreover, Brazil is the largest producer and exporter of tropical forage seeds in the world [2].

One of the most widely cultivated species of *Urochloa* is *Urochloa decumbens* Stapf., particularly *U. decumbens* cv. ‘Basilisk’. This species exhibits exceptional adaptation to the poor and acidic soils that are typical of the tropics and lead to good animal performance [4]. However, the molecular genetic information regarding this species is limited, mainly due to its reproducing predominantly via apomixis and because its ploidy levels range from diploid to pentaploid [5].

The need for new more productive and efficient cultivars has inspired the search for new tools to facilitate the selection process [3]. Thus, genetic and genomic studies are essential to advancing breeding programs via a better understanding of the genetic structure of the species. These types of studies can be conducted by using molecular tools, such as molecular markers.

Among all molecular markers, one of the most effective for plant genetics studies is the microsatellite, also known as the SSR (Simple Sequence Repeat). These markers are highly informative due to their multi-allelic nature, co-dominant inheritance, high transferability and broad distribution in the genomes of the species [6–8].

Whereas some microsatellite markers for *Urochloa* species have been developed [9–13], specific microsatellite markers for *U. decumbens* have not been reported. Specific microsatellite molecular markers can be very useful in assessing the genetic diversity of germplasms, performing linkage mapping, identifying quantitative trait loci (QTL), performing genome-wide selection and marker-assisted selection, and facilitating molecular based breeding to improve the economically importance characteristics of a species [6, 7]. Moreover, microsatellite markers identified in species with little genome information may be used for cross-amplification between related species [14].

The aims of the present study were to identify and characterize the first set of microsatellite markers for *U. decumbens* and to test their transferability to four other *Urochloa* species (*U. brizantha, U. dictyoneura, U. humidicola* and *U. ruziziensis*).

## Methods

Thirty-four *Urochloa* genotypes were obtained from the Embrapa Beef Cattle collection, in Campo Grande, MS, Brazil for marker validation. Twenty of these genotypes are represented by *U. decumbens* accessions, six genotypes are intra-specific hybrids of the same species and the other eight genotypes are represented by two different germplasm accessions each from *U. brizantha, U. humidicola, U. dictyoneura* and *U. ruziziensis*. These other *Urochloa* species were used for the cross-amplification tests. The annotation numbers, accession numbers (as recorded in the Embrapa- BRA-, in the Embrapa Beef Cattle- EBC- and in the Center for Tropical Agriculture- CIAT- databases), genotypes, species identified, their mode of reproduction and the origin of the genotypes are shown in Table 1.

**Table 1:** Genotypes of *U. decumbens* and four other species of the genus *Urochloa* that were used to characterize the microsatellite markers and analyze their levels of transferability

AN	CIAT	BRA	EBC	Origin	MR	Genotype	Species
1	16494	004448	D005	Kenya	SEX	Germplasm accession	*U. decumbens*
2	16495	004456	D006	Kenya	SEX	Germplasm accession	*U. decumbens*
3	16497	004472	D007	Kenya	APO	Germplasm accession	*U. decumbens*
4	16498	004481	D008	Kenya	APO	Germplasm accession	*U. decumbens*
5	16499	004499	D009	Kenya	APO	Germplasm accession	*U. decumbens*
6	16500	004502	D010	Kenya	APO	Germplasm accession	*U. decumbens*
7	16501	004511	D011	Kenya	APO	Germplasm accession	*U. decumbens*
8	16504	004545	D014	Kenya	APO	Germplasm accession	*U. decumbens*
9	26295	004651	D024	Rwanda	SEX	Germplasm accession	*U. decumbens*
10	26300	004707	D028	Rwanda	APO	Germplasm accession	*U. decumbens*
11	26304	004740	D032	Rwanda	APO	Germplasm accession	*U. decumbens*
12	26308	004782	D035	Rwanda	SEX	Germplasm accession	*U. decumbens*
13	16491	004421	D036	Kenya	APO	Germplasm accession	*U. decumbens*
14	26306	004766	D040	Rwanda	SEX	Germplasm accession	*U. decumbens*
15	6370	000116	D059	Unknown	APO	Germplasm accession	*U. decumbens*
16	16100	001961	D061	Unknown	APO	Germplasm accession	*U. decumbens*
17	NA	001996	D070	Unknown	APO	Germplasm accession	*U. decumbens*
18	6298	000060	D077	Unknown	APO	Germplasm accession	*U. decumbens*
19	–	–	D024/27	CNPGC	SEX	Tetraploidized accession	*U. decumbens*
20	606	001058	D062	Uganda	APO	Germplasm accession	*U. decumbens*
21	–	–	R10	CNPGC	NA	Hybrid	*U. decumbens*
22	–	–	R44	CNPGC	APO	Hybrid	*U. decumbens*
23	–	–	R125	CNPGC	NA	Hybrid	*U. decumbens*
24	–	–	R144	CNPGC	APO	Hybrid	*U. decumbens*
25	–	–	R146	CNPGC	NA	Hybrid	*U. decumbens*
26	–	–	R182	CNPGC	NA	Hybrid	*U. decumbens*
27	16186	007889	DT157	Ethiopia	APO	Germplasm accession	*U. dictyoneura*
28	16188	007901	DT159	Ethiopia	APO	Germplasm accession	*U. dictyoneura*
29	NA	NA	R044	Unknown	SEX	Germplasm accession	*U. ruziziensis*
30	26163	005568	R102	Burundi	SEX	Germplasm accession	*U. ruziziensis*
31	16125	002844	B112	Ethiopia	APO	Germplasm accession	*U. brizantha*
32	26110	004308	B178	Burundi	APO	Germplasm accession	*U. brizantha*
33	26149	005118	H016	Burundi	APO	Germplasm accession	*U. humidicola*
34	6369	000370	H126	Unknown	APO	Germplasm accession	*U. humidicola*

*AN* annotation number, *CIAT* Center for Tropical Agriculture, *BRA* codes from Embrapa, *CNPGC* National Center for Research on Beef Cattle, *EBC* codes from Embrapa Beef Cattle, *MR* mode of reproduction- apomictic or sexual, *NA* not available

Genomic DNA was isolated from fresh leaves using the CTAB method [15]. The purity and concentration of the isolated DNA were determined using a NanoDrop1000 (Thermo) spectrophotometer and by electrophoresis in a 0.8 % agarose gel that was subsequently stained with ethidium bromide (5 µg/mL^−1^).

In a previous study, a microsatellite-enriched library of one *U. decumbens* genotype was constructed using the method described by Billotte et al. [16]. The sequences were then treated as described previously [9]. The microsatellites were identified using MISA software [17], and only mononucleotides with 12 or more repeats, dinucleotides with six or more repeats, trinucleotides with four or more repeats, and tetra, penta, and hexanucleotides with three or more repeats were considered. The DNA sequences determined in this study were deposited in GenBank under the accession numbers shown in Table 2.

**Table 2:** Description of the 93 SSR markers developed for *U. decumbens*

SSR locus	GenBank accession number	Primer sequences (5′–3′)	Repeat motif	Ta (°C)^a^	Size (bp)	NA^b^	PIC^c^	DP^d^
Dec01	KT587691	F_CAAACGACTGCTGATGATGG	(AC)_16_	65°	250–280	5	0.68	0.89
		R_TGAGAGGCTAAGAG/CAACCTG						
Dec03	KT587692	F_AACTGAACGCTGCTTGGTCT	(GT)_6_	65°	240–260	3	0.58	0.63
		R_GGTCCGGAATAAAAAGCACA						
Dec05	KT587693	F_GGGCTCCTCATCAGCAGTAG	(GAC)_4_	65°	132–140	4	0.61	0.54
		R_GATGCCTCTCGGGACTATCA						
Dec06	KT587694	F_GTTCATGGGGGCAATCAGT	(CTGG)_3_	65°	120–130	4	0.70	0.54
		R_CGTGATGTCTGAACGGATGA						
Dec07	KT587695	F_CGAACACATTCACATACAACA	(AC)_7_	65°	226–242	5	0.74	0.87
		R_CTGTCGGATTTATTTGCATTA						
Dec09	KT587696	F_GCCCAACTGGAATGTGCTA	(TC)_9_	65°	240–280	5	0.72	0.91
		R_CGACGTCCTTGTTGTTTGTC						
Dec10	KT587697	F_GACGTCGAGGACAAACAACA	(CAAG)_3_	65°	216–256	6	0.79	0.86
		R_TCCTTACCCTTGCGATTCAC						
Dec11	KT587698	F_GGGGGAAAATGAGACAGACA	(AG)_16_	65°	154–198	8	0.80	0.94
		R_GCTAACCAGACAGCCACCAC						
Dec12	KT587699	F_CTCACACCCTCCTTCTGCTG	(GT)_9_	65°	196–226	9	0.82	0.97
		R_CGATCGCTCCCTACTAGTGC						
Dec13	KT587700	F_CCCCCGTAAAACAGACAAAA	(TA)_6_	65°	166–178	5	0.72	0.89
		R_ACCATGATACAACGCTGCAA						
Dec14	KT587701	F_AAACGGAGAAAGGGGATCAT	(GAC)_4_	65°	290–310	3	0.62	0.22
		R_GAGCATACATGCAGCAGTGG						
Dec17	KT587702	F_CCTTCGTCCATTACCCTGAA	(TG)_9_	65°	224–248	6	0.63	0.72
		R_ATCCACCAGTGCACGTATGA						
Dec18	KT587703	F_ACGCACACACACGAACAAAT	(CGAT)_3_	65°	180–202	6	0.78	0.96
		R_ATTTCGACATGCCTGCAACT						
Dec19	KT587704	F_AGGTTCGATAATCGGCACAC	(GT)_7_	65°	220–236	6	0.79	0.95
		R_CGCAAGTGGTCAAGCAATTA						
Dec20	KT587705	F_ACCTTGAACTCCTGCTTTTGT	(AC)_10_	65°	150–168	6	0.75	0.92
		R_AGCACTATCACCAATCAGCAA						
Dec21	KT587706	F_GCCGACATCAACTTCCATTT	(GT)_7_	65°	176–190	5	0.76	0.85
		R_CTCCTTGGTCCAATTCCTCA						
Dec22	KT587707	F_GTGTGTACGTGATGCTATGTG	(CTT)_4_	65°	186–192	4	0.47	0.57
		R_ATCGATCTCACTGACCATGT						
Dec24	KT587708	F_TAAAGAAACATGGGCCGGTA	(GCC)_5_	65°	210–226	5	0.73	0.86
		R_TTATTCCTGGGATTGGGTTG						
Dec26	KT587709	F_TCGGAAAACGCAGGAGAG	(CA)_6_	65°	180–190	4	0.68	0.59
		R_GTTCAGTGGGTCTGGCTTGT						
Dec27	KT587710	F_TGTACATGAATGGCAGCACA	(AGAT)_3_	65°	248–262	6	0.73	0.76
		R_AACAGCAGCAGAGATGACGA						
Dec28	KT587711	F_GTTCCTCCCAAGAAACCACA	(AC)_6_	65°	146–180	8	0.78	0.84
		R_CCCAACATTCACCTGGTTCT						
Dec29	KT587712	F_TGTTATAATCATCACCATGCTC	(GTA)_4_	65°	170–184	6	0.70	0.67
		R_ACAGCTATTGCCACTACTTGA						
Dec30	KT587713	F_CATTACGAGCACGCAGTCC	(CA)_7_	65°	152–164	5	0.71	0.59
		R_TACCACTGCTGGACACGAGA						
Dec31	KT587714	F_CGTTGTCAGCACACACACAC	(TCTA)_3_	65°	136–146	5	0.70	0.79
		R_TACTACCACTGCTGGACACGA						
Dec33	KT587715	F_TGTCGTGTGCGTTTTGTTTT	(CTT)_4_	60°	274–336	8	0.78	0.94
		R_CTAAGATCCCCACTCCCACA						
Dec35	KT587716	F_TTCTTGGACACACAGCCTTG	(TG)_4_	65°	274–290	6	0.72	0.88
		R_GGGCTGAAAACATCATCACC						
Dec36	KT587717	F_GAAGGTGATGATCGGCAGTT	(GCAG)_3_	65°	280	1	0.00	0.00
		R_GTGTGCGTTGCTGCCTACTA						
Dec37	KT587718	F_CCTCTCTTCCGTTTGCTCTG	(GTG)_5_	65°	198–218	5	0.70	0.81
		R_TGAACAGGCACGGATTGATA						
Dec39	KT587719	F_TAGGTGTCCCATTGGTCGAT	(GT)_7_	55°	166–182	5	0.64	0.34
		R_AGGAGAGCTGCGTGTCATTT						
Dec42	KT587720	F_CACGTCATGTACTGCGATCC	(GT)_6_	65°	220–230	3	0.56	0.68
		R_GCGTCACACATACACACACG						
Dec43	KT587721	F_CAGTCATCAGCATTCAGGTAT	(TG)_11_(AG)_6_	65°	212–228	5	0.74	0.91
		R_ATAACTTGCGTATGTGCTCTC						
Dec44	KT587722	F_CATGCTTAATCCAGAAATCAG	(AC)_12_	65°	182–226	6	0.78	0.94
		R_TGTAAACCGGAAAGTGTACTG						
Dec45	KT587723	F_TGGAGATGGAGATGGGAGTC	(GGAT)_3_	65°	210	1	0.00	0.00
		R_CCCAAGGAATGGGATAGGTT						
Dec47	KT587724	F_AGAGAGCTGATGGTCGTGGT	(GA)_9_	65°	210	1	0.00	0.00
		R_TGGAAACTTGGGAGGATCTG						
Dec48	KT587725	F_CTAACGCTATTGCTTTGCTT	(CT)_45_	65°	144–190	10	0.85	0.94
		R_TGCAGAGAGAGAGAAGAGAGA						
Dec49	KT587726	F_CAATGCATGCTTGGAACTTG	(GT)_6_	65°	166–180	5	0.65	0.74
		R_CATCGGAGGGTAGATTGGTC						
Dec50	KT587727	F_GAAACAGGACCATCAGATAGCA	(CA)_6_	65°	164–180	5	0.76	0.84
		R_GGAATCTGCAGGTTTGGAAG						
Dec51	KT587728	F_GCTGATCCTCGGATTGTGTT	(TG)_21_	65°	248–262	5	0.69	0.92
		R_TAACTTGGACGCGCTAAAGG						
Dec52	KT587729	F_CACGAATGCACATGCAATAA	(GT)_6_	65°	289–292	2	0.00	0.00
		R_AGTGAACCAAACTGCCAGAA						
Dec54	KT587730	F_GCCCTCTTTAACTCTGCTTTA	(CA)_8_	65°	236–252	5	0.75	0.92
		R_GTATCTTCTTTCGGATGACCT						
Dec55	KT587731	F_AGCACCATCATCTTTAACAAA	(ACACC)_3_	65°	212–224	6	0.78	0.73
		R_CAAGGAATTTGCACTAAAAGA						
Dec56	KT587732	F_GAACTTAATGGCGGAGTAGAC	(AG)_14_	55°	220–230	2	0.00	0.00
		R_CACAGATTGCTGAATTGTTTC						
Dec58	KT587733	F_ATTAGGATTGCGCACTGGTC	(GT)_6_	65°	286–298	5	0.64	0.8
		R_ATCCGCATTCACAACCTCTC						
Dec59	KT587734	F_GGTTAAAATGGTTCGCTGGA	(GT)_7_	65°	184–220	5	0.73	0.92
		R_ACCTAGGCTCGCATGACAAT						
Dec60	KT587735	F_ATTTCAGTTGCACATTCCA	(GT)_6_	55°	220–230	2	0.00	0.00
		R_TCCAAAACTTAGCTCAGAAAG						
Dec62	KT587736	F_AGGAAGGGTACGGTGTAGGC	(CA)_7_	65°	216–238	4	0.41	0.59
		R_TCTACATGCACATCCGGAAA						
Dec63	KT587737	F_GGGATATTTTCCGGATGT	(CTT)_4_	65°	218–226	3	0.51	0.7
		R_CAGAGCTCAGAAAGTCGTTAC						
Dec65	KT587738	F_TCGGATTCTTGGACAACCTC	(GGCC)_3_	65°	180	1	0.00	0.00
		R_CCTCTACGCGAAAGATGGTC						
Dec69	KT587739	F_GATGGCTACCTGCATTGGAT	(CCAT)_3_	55°	168–180	6	0.79	0.96
		R_ATAAGGGGAGCCCTCAAAAA						
Dec70	KT587740	F_AGCTGCCTCCACTTGACAAT	(TG)_7_	65°	256–268	5	0.72	0.62
		R_AGGCCCTGATAGTCCCCTAA						
Dec71	KT587741	F_GAGCTTCCCTGTGTCTGATA	(TG)_10_	55°	234–254	4	0.62	0.84
		R_ATGACAATGACTATGCTGACC						
Dec75	KT587742	F_ACAGGAGCCTTTATGCATGG	(ATGC)_3_	65°	150–166	5	0.68	0.69
		R_GTCCTGTGTTGGTCGTTCCT						
Dec76	KT587743	F_GTCACGTGCCATCACAAATC	(TAGC)_3_	65°	270	1	0.00	0.00
		R_GCACACATGCATGATGACAA						
Dec77	KT587744	F_TCCAAATGTACCGTCAATAAA	(AG)_12_	55°	234–260	7	0.76	0.9
		R_CGTGTCTGCATTCAAAGTG						
Dec78	KT587745	F_GCTTACCACATCCGGTGATT	(AC)_8_	65°	246–260	5	0.66	0.71
		R_GAGAATGCTTCCCGTTCTTG						
Dec83	KT587746	F_GGCTTGCTCCAAGAGATGAG	(CA)_20_	65°	174–198	4	0.66	0.72
		R_TAGCTTGGCCTTTGTGTGTG						
Dec84	KT587747	F_GGCTTGCTCCAAGAGATGAG	(AC)_9_	65°	220–250	7	0.78	0.95
		R_TTCGTCACGTCAAAACAAGC						
Dec86	KT587748	F_CCACCTCCCAGGATAGATGA	(TG)_7_	55°	140–180	9	0.80	0.94
		R_AGATTGGGGGAGGAAGAAGA						
Dec89	KT587749	F_CTGTTGCATCCACCACTTTTT	(TC)_8_	65°	146–180	4	0.55	0.41
		R_CGGCAGCCTAAAGTGATTGT						
Dec90	KT587750	F_CGGTGCTCCATGATTAGGAT	(GT)_8_	65°	278–326	7	0.77	0.82
		R_GCGTAGCATCATCGAGAACA						
Dec91	KT587751	F_GCCTCATCTGTTCATTCATT	(TG)_7_	55°	290–330	3	0.26	0.22
		R_TGGCACTCTAACTTGTAGGC						
Dec92	KT587752	F_AGCAATCCAAGCTGAAAGGA	(AC)_7_	65°	264–290	7	0.79	0.92
		R_TTCCGCATGAAACAAAACTG						
Dec93	KT587753	F_TTCGGTCAAAATCGAAAAGG	(AC)_6_	65°	226–244	5	0.72	0.95
		R_GCATTGTTTCAGAGGCTTCG						
Dec95	KT587754	F_AGCAACCCAAAGGTCAGCTA	(CT)_24_	65°	178–208	6	0.71	0.89
		R_AGGAGGGATTCAAGGGAGAA						
Dec96	KT587755	F_CATTCTGGTATGGCACGTTG	(CA)_6_	65°	148–154	4	0.66	0.85
		R_ATTTACCGACCAGGCTGAAG						
Dec97	KT587756	F_GGGCAGGCACTAGATTGATT	(TCTT)_3_	65°	176–184	4	0.61	0.72
		R_TTGCTTGCTTGAGTTTGTGG						
Dec98	KT587757	F_TAGGTGACAAGGCACGATCA	(AG)_10_	65°	252–272	7	0.76	0.95
		R_GGGCCAACATACCAAAGAGA						
Dec99	KT587758	F_TAAGAGACGAGTGCTCTGAAA	(AGCAGG)_3_	65°	210–228	7	0.77	0.91
		R_TTGTGAATCGGTACTTTTGTC						
Dec101	KT587759	F_CTCTAACTTTCGGCGTGGTC	(GGCC)_3_	65°	224–230	3	0.53	0.71
		R_GGACGGTCCGACTTGTCTAA						
Dec103	KT587760	F_ATGACGAACTTGCTCCCTACA	(AC)_8_	55°	176–206	4	0.51	0.71
		R_ATCGATTCAGAGCCGCTTC						
Dec105	KT587761	F_CCTTCTGTTCATTGCAGTCC	(TG)_8_	65°	174–180	4	0.56	0.65
		R_TGGTACCACAATGCCAAATC						
Dec106	KT587762	F_TCACGAACAACGATCAGAGC	(TG)_7_	55°	180–230	7	0.74	0.93
		R_TCTTTACCCGTGCTGTTTCC						
Dec108	KT587763	F_CATCACCGCATTTATGCAAG	(AG)_8_	65°	184–200	6	0.68	0.85
		R_ACACACGTCCTCGTCTTCCT						
Dec109	KT587764	F_CAGCACACTGAATCCTCTGC	(GT)_6_	65°	216–220	3	0.39	0.59
		R_CCGTTGTTCCATCAGAACCT						
Dec110	KT587765	F_CTCCGAAGATCCGAGCTATG	(GT)_7_	65°	178–184	4	0.31	0.41
		R_CCCCTGGAGGCTATAAAAGG						
Dec111	KT587766	F_TGATTAGGTGCTGACTGCTG	(ATTT)_3_	65°	178–186	5	0.50	0.57
		R_CTGGAAGATGTATTTGGTGTGA						
Dec112	KT587767	F_CCTCAAGAAGCTCTGGGATTT	(TGTT)_3_	55°	238–244	4	0.57	0.72
		R_TGTGCAAACGTCAGTAGAGCA						
Dec113	KT587768	F_TGGACTAACTGCACTGCCTGT	(GT)_9_	65°	208–224	7	0.74	0.94
		R_CATGAGGAGCACAGCGAATA						
Dec114	KT587769	F_CAAAGGCCATGCCTTGTACT	(GT)_11_	65°	214–220	4	0.62	0.72
		R_CACTGCTCAGCCAATCCTAAG						
Dec115	KT587770	F_GGCATATGTCTGAGTAAGTGTG	(TCT)_4_	55°	160–174	6	0.76	0.6
		R_CCTGTTTCCATTGATTCTTTT						
Dec116	KT587771	F_TCACTTCATCCATTCGCTTG	(TG)_17_	65°	274	1	0.00	0.00
		R_AACATGACCGACTCCTACGG						
Dec118	KT587772	F_ACACACCCCAACTCACACAA	(AC)_6_	65°	208–226	6	0.75	0.83
		R_TGGTCATGGCAAAAGATGAA						
Dec121	KT587773	F_TGCACAATGATGAACACAGG	(GT)_7_	65°	226–264	6	0.74	0.74
		R_AGTGAACCAAACTGCCAGAA						
Dec122	KT587774	F_CCTGCGTCACTCGAGAAAA	(TCTG)_3_	65°	268–292	6	0.76	0.93
		R_CAATGTCATCGCCATTTCTG						
Dec123	KT587775	F_TGAGCAACACTGGAGAATGG	(TC)_9_	65°	248–280	9	0.80	0.94
		R_CGTACATGACAGGAGGGTGTT						
Dec124	KT587776	F_AGAAGCCCCAGATGTTCTGA	(GT)_9_	65°	270–306	4	0.52	0.69
		R_GCTAGTCGCGTCTACCGTTC						
Dec125	KT587777	F_TCTGGGGTGGAAATGTTGAT	(CT)_11_	65°	202–214	4	0.61	0.34
		R_CCCTTCACCTTGAGAAAGCA						
Dec126	KT587778	F_GGATGGATTGATGGATGCTT	(GGCC)_3_	65°	268–304	7	0.77	0.93
		R_AACCCGAAAGGCCTAAGCTA						
Dec127	KT587779	F_CGTTGATCACACGTCTCAGG	(TTGC)_3_	65°	250–280	4	0.65	0.75
		R_GATTTCGCCACCAACATTCT						
Dec131	KT587780	F_CTTGTTACCTTCTGCACAATAAA	(GAA)_5_	65°	160–170	3	0.00	0.00
		R_ATTAGTCTTTCCGTCCTTGTC						
Dec132	KT587781	F_GTATCGGGTAGCAAGGCAAG	(AAGC)_3_	65°	220–240	2	0.00	0.00
		R_GGAAATTCCTTACCCCGAAG						
Dec133	KT587782	F_GGATGGAAGAGCACAAAAGC	(CT)_7_	65°	218–228	5	0.68	0.81
		R_GCGTGTGTGTGTGTGTTTGA						
Dec134	KT587783	F_CAGGCTTCCCCTCTCTCTCT	(AC)_7_	65°	220–260	8	0.76	0.93
		R_GCAACCGGAAGAATTCATGT						
Total average						4.93	0.68	0.77

^a^Amplification temperature (°C)

^b^Maximum number of alleles observed

^c^Polymorphism information content

^d^Discrimination power

After the primer pairs were designed using Primer3Plus software [18], we added a M13 tail (5′CACGACGTTGTAAAACGAC-3′) to each forward primer. Polymerase chain reaction (PCR) assays were conducted as described previously [9]. The amplified products were separated by electrophoresis through 3 % agarose gels prior to vertical electrophoresis through 6 % denaturing polyacrylamide gels. The gels were then silver stained [19], and the product sizes were determined by comparison to those of a 10 bp DNA ladder (Invitrogen, Carlsbad, CA, USA).

We considered only the strongest bands because the less intense bands might have been stutter bands and an SSR was considered transferable when a band of the expected size was amplified via PCR and an appropriate SSR pattern was observed. Each SSR allele was treated as dominant due to the high ploidy levels of the genotypes; thus, this analysis was based on the presence (1) or absence (0) of a band in the polyacrylamide gels.

The genetic distance among the genotypes was evaluated according to Jaccard’s coefficient [20] based on a binary matrix constructed using the molecular data. This analysis was conducted using the software package NTSYSpc 2.11X [21]. An unrooted tree was constructed using the weighted neighbor-joining method (NJ) using DARwin 6.0.010 software [22].

The set of molecular data was also analyzed using the admixture model of STRUCTURE software version 2.3.4 [23] to infer the population structure of the 34 genotypes. The admixture model was tested using a period of burn-in with 100,000 iterations and a run length of 200,000. The number of K (clusters) was set from 2 to 20. To infer the appropriate number of clusters in our data, we used the ΔK statistic, which represents the rate of change in the log probability of the data between successive K values rather than the log probability of the data [24]. We retained the K value corresponding to the highest value of ΔK obtained using the online tool Structure Harvester [25].

The polymorphism information content (PIC) values were calculated to evaluate the levels of marker informativeness and to help choose primers for future studies [26]. To compare the efficacies of the markers used for varietal identification, the discrimination power (DP) value was determined for each primer [27].

## Results

We analyzed 281 contigs, of which 128 were found to contain SSR. One hundred fifty-five SSR motifs were found, with the perfect microsatellite being the most abundant. Dinucleotide repeats were the most abundant class of microsatellite detected (59.36 %), followed by tetranucleotide (18.71 %), trinucleotide (12.26 %), mononucleotide (3.87 %), hexanucleotide (3.22 %) and pentanucleotide (2.58 %) repeats. Furthermore, 22 % of the microsatellite motifs were classified as class I motifs (>20 bp), and 78 % were classified as class II motifs (from 12 to 20 bp).

A total of 113 specific primer pairs were designed, and 93 SSR markers amplified from *U. decumbens*, with 82 of these being polymorphic. A total of 459 bands were scored, and the number of bands per locus was found to range from 1 to 10, with an average of 4.93 bands per locus (Table 2).

The PIC values of the 82 polymorphic loci ranged from 0.26 to 0.85 (average of 0.68), and the discrimination   power (DP) values ranged from 0.22 to 0.97 (average of 0.77) (Table 2).

Two genotypes of four other species of the genus *Urochloa* (*U. brizantha, U. humidicola, U. dictyoneura* and *U. ruziziensis*) (Table 1) were used to evaluate the transferability of the 93 SSR markers. All of the loci were tested using the same PCR conditions used for analysis of *U. decumbens*. Fifty-six percent of the loci were amplified in at least one *U. dictyoneura* genotype, 38 % were amplified in *U. humidicola*, 99 % were amplified in *U. ruziziensis*, and 92 % were amplified in *U. brizantha.* Amplification of 33 % of the microsatellite markers was achieved for all of the evaluated species. The microsatellite markers Dec07, Dec31, Dec33, Dec77 and Dec108 were only transferable for *U. ruziziensis* species (see Additional file 1).

Based on the allelic frequencies determined using STRUCTURE software [23], 28 % of the alleles are rare (frequency < 0.05), 57 % of these alleles are of intermediate abundance (0.05 < frequency < 0.30), and 15 % are abundant alleles (frequency > 0.30). We observed 43 rare alleles that are specific for *U. decumbens*, eight rare alleles specific for *U. humidicola*, seven specific for *U. dictyoneura*, four alleles specific for *U. brizantha* and two rare alleles specific for *U. ruziziensis*.

**Fig. 1:**
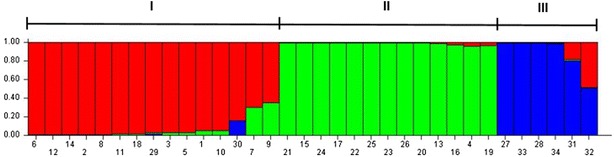
Analysis performed using an admixture model in STRUCTURE 2.3.4 software with correlated allele frequencies. The clustering profile obtained at K = 3 is indicated by different *colors*. Each of the 34 genotypes is represented by a single column broken into colored segments with lengths proportional to each of the K inferred gene pools. The scale on the *left* indicates the membership coefficients (Q) used to allocate the genotypes into clusters. The genotypes were named according to the annotated numbers listed in Table 1. Cluster I (from 6 to 9), Cluster II (from 21 to 19) and Cluster III (from 27 to 32)

The Bayesian analysis performed using STRUCTURE software [23] revealed that the 34 *Urochloa* genotypes could be distributed into three distinct clusters (Fig. 1), as determined from the ΔK values that were generated using Structure Harvester software [24, 25] (see Additional file 2). Using a K value of three, 15 genotypes were allocated into Cluster I (6 to 9), 13 genotypes were grouped into Cluster II (21 to 19) and six genotypes were allocated into Cluster III (27 to 32) (Fig. 1).

**Fig. 2:**
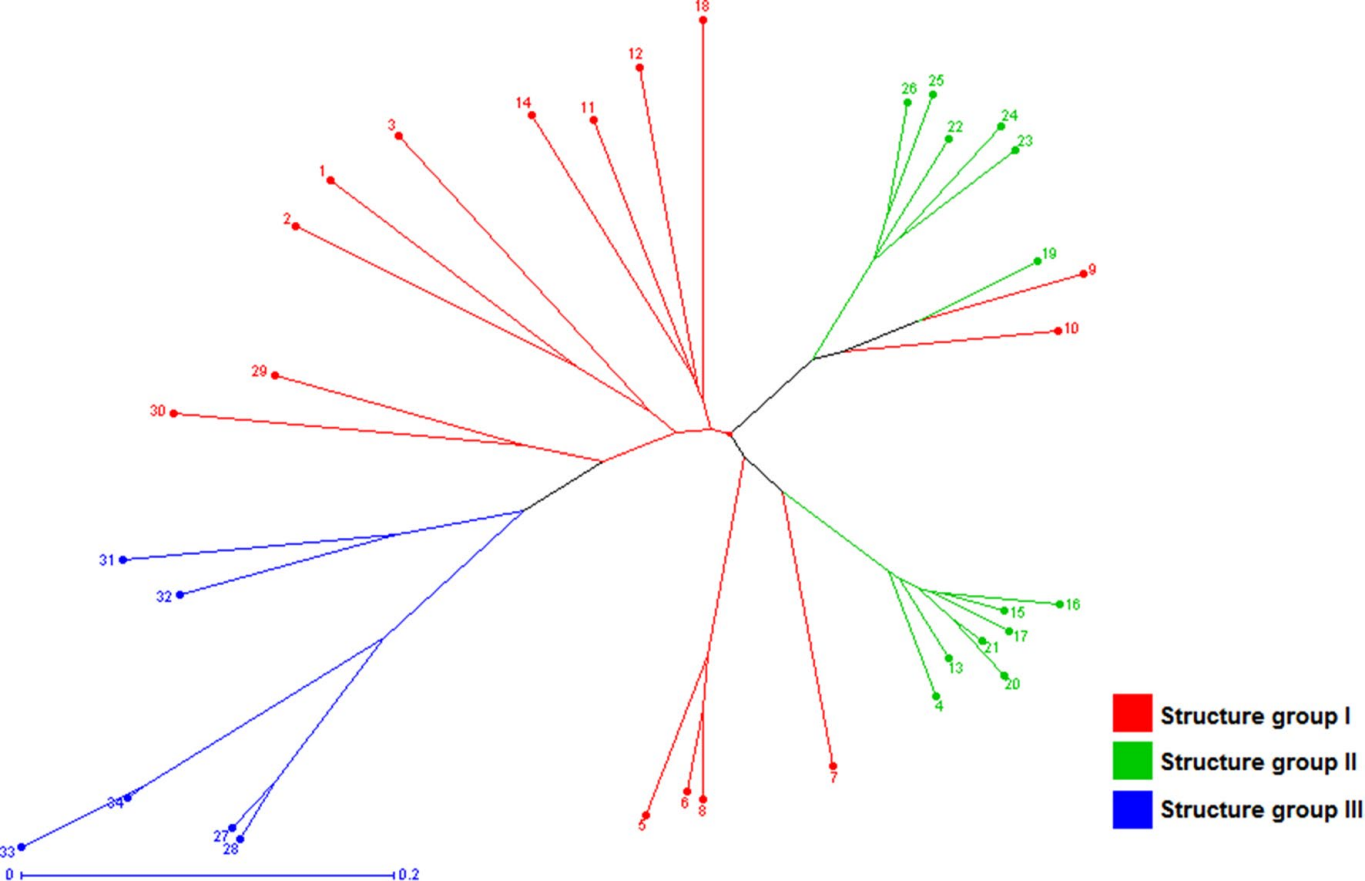
Unrooted neighbor-joining tree based on Jaccard’s coefficient for the 34 genotypes of the *Urochloa* species. The genotypes were named according to the annotated numbers listed in Table 1. The *colors* of the branches represent the clusters identified in Fig. 1, as follows: *red* Cluster I; *green* Cluster II; *blue* Cluster III

The genetic distance values that were determined using Jaccard’s coefficient ranged from 0.06 (D062 and R10) to 0.76 (H016 and D009) (see Additional file 3). The unrooted neighbor-joining tree successfully discriminated all of the tested genotypes (Fig. 2).

## Discussion

In this report, we have described the first set of microsatellite markers for *U. decumbens*, which is an important tropical forage grass for which there is limited genetic information. The availability of a robust set of informative molecular markers is essential to accelerating its breeding programs as well as for germplasm characterization, genetic map development and marker-assisted selection.

In the present study, dinucleotide repeats were the most abundant class of microsatellites detected, followed by tetra, tri, mono, hexa and pentanucleotide repeats. Dinucleotide motifs have been found to be the most abundant type of microsatellites in plant genomes [28, 29]. Notably, the high occurrence of dinucleotide motifs can be attributed to both of the evaluated libraries having been enriched using (CT)_8_ and (GT)_8_ probes.

In total, 93 SSR markers were characterized, 82 of which were found to be polymorphic (88 %). The loci that did not exhibit polymorphism in the genotypes that we evaluated may be useful in other studies.

The Polymorphism Information Content (PIC) is an index used to qualify a marker for genetic studies and reflects the level of polymorphism detected. Seventy-seven markers tested in *U. decumbens* genotypes were found to be highly informative (PIC > 0.5) and five markers were found to be moderately informative (0.25 < PIC < 0.5), based on a previously proposed classification system [30] (Table 2). The Dec48 marker had the highest PIC value, 0.85, and the Dec91 marker had the lowest value, 0.26. The average PIC values for all of the markers was 0.68 (Table 2), indicating a high level of polymorphism.

To determine whether these molecular markers could discriminate the genotypes of *U. decumbens*, the discrimination power (DP) of each SSR locus was computed. The PD values ranged from 0.22 (Dec14 and Dec91) to 0.97 (Dec12), with an average value of 0.77.

The most informative loci in this panel of SSRs were Dec12, Dec48, Dec86 and Dec97 because they had the highest PIC and DP values (Table 2). In contrast, the Dec91 locus had low PIC and DP values (0.26 and 0.22, respectively), as expected due to its low levels of polymorphism and cross- amplification in all of the other *Urochloa* species tested, which suggests that this locus is a conserved region [11].

Structure analysis showed that the genotypes were distributed in three clusters and that each cluster was characterized by a set of allele frequencies at each locus and was represented by different colors (red, green and blue) as shown in Fig. 1. The best K number of clusters was determined using the ΔK method [24] and implemented in the online tool Structure Harvester [25] (see Additional file 2).

Cluster I included fifteen *U. decumbens* genotypes plus the *U. ruziziensis* genotypes, Cluster II contained only *U. decumbens* genotypes, and Cluster III contained the others *Urochloa* species, including *U. dictyoneura, U. humidicola* and *U. brizantha* (Fig. 1). The clustering of some of the *U. decumbens* genotypes with *U. ruziziensis* genotypes may be explained by the genetic proximity of these species [11, 13, 31, 32]. This fact is reflected in the allelic pools that are identified with different colors in Fig. 1.

Cluster II included genotypes 19 and 20, and six hybrids derived from crosses between these two genotypes that were grouped together (Fig. 1). These hybrids are members of an F_1_ population that will be mapped using the polymorphic SSRs described in this study. In Cluster III, which included three different *Urochloa* species, the predominant allelic pool is represented in blue, and only the *U. brizantha* genotypes showed some percentage of the red allelic pools, demonstrating their genetic proximity to *U. decumbens* (Fig. 1).

The tree constructed based on Jaccard’s coefficient successfully discriminated all of the tested genotypes (Fig. 2) and showed a distribution of these genotypes similar to that obtained using STRUCTURE software [23] (Fig. 1), although the two types of analysis used different statistical approaches. Moreover, this tree and the allelic pools that were determined indicated that *U. decumbens* and *U. ruziziensis* are more closely related to one another than to the other species (Figs. 1 and 2).

Based on the genetic values obtained using Jaccard’s coefficient, the lowest genetic distance was observed between the D062 and R10 genotypes (0.06). The R10 genotype should correspond to a hybrid that originated from a cross between D062 and D24/27, but the genetic distance observed shows that it is likely a false hybrid, which demonstrates the importance of using molecular markers to discriminate genotypes. The highest genetic distance (0.76) was observed between the D009 and H016 genotypes, representing *U. decumbens* and *U. humidicola* species, respectively, which are genetically distant species [11, 13, 31, 32] (see Additional file 3).

All of the microsatellite markers were transferable to at least one different species of the *Urochloa* genus, and 33 % of the markers were successfully amplified in all of the species, indicating their absolute transferability. The highest level of transferability was observed in *U. ruziziensis*, followed by *U. brizantha*, *U. dictyoneura* and *U. humidicola* (see Additional file 1). The higher proportion of successful PCR amplification for the *U. ruziziensis* and *U. brizantha* genotypes indicates the closer phylogenetic distance between these species and *U. decumbens*. Thus, *U. brizantha, U. decumbens* and *U. ruziziensis* form an agamic complex and produce fertile hybrids [33, 34], enhancing the *Urochloa* breeding program.

Silva et al. [12] developed 198 polymorphic microsatellite markers for *U. ruziziensis* and found that the percentages of markers potentially transferable to *U. decumbens* and *U. humidicola* were 92.9 % and 42.9 %, respectively, corroborating our results. Others studies showed that *U. brizantha* and *U. ruziziensis* are more closely related to *U. decumbens* than to *U. humidicola* and *U. dictyoneura* [11, 13, 31, 32]. Marker transferability is effective in reducing the time and cost of initial studies aimed at identifying microsatellite markers in related species; thus, these markers could be used in genetics studies, such as in those concerning intra-species molecular characterization, species differentiation, molecular identification, and characterization of interspecific hybrids [14].

The success of a breeding program can be accelerated by the effective use of molecular markers. Thus, the SSR markers developed in this study will be useful for *U. decumbens* breeding programs and possibly for those of other related *Urochloa* species.

## Availability of supporting data

The datasets supporting the results of this article are included in the article.

## Additional files

10.1186/s13104-016-1967-9 Transferability of the SSR markers developed for *Urochloa decumbens*.
10.1186/s13104-016-1967-9 Magnitude of ΔK determined in STRUCTURE analysis of K, calculated following the ΔK method proposed by Evanno et al. [24].The highest ΔK value corresponds to the optimal K.
10.1186/s13104-016-1967-9 Jaccard’s coefficient for 34 genotypes of *Urochloa* spp. evaluated using 82 microsatellite markers. Individuals are identified according to their EBC codes (Table 1).

## Abbreviations

AN: annotation number
bp: base pairs
CAPES: Coordination of Improvement of Higher Education Personnel
CTAB: cetyltrimethyl ammonium bromide
DNA: deoxyribonucleic acid
DP: discrimination power
EBC: Embrapa Beef Cattle
EMBRAPA: Brazilian Agricultural Research Corporation
K: number of clusters
MCMC: Markov Chain Monte Carlo
NA: number of alleles
NJ: neighbor joining
PCR: polymerase chain reaction
PIC: polymorphism information content
Q: association coefficient determined using STRUCTURE analysis
QTL: quantitative trait loci
SSR: simple sequence repeat
Syn: synonym
Ta (°C): annealing temperature
