# Supplementary material for: Microsatellite loci for Urochloa decumbens (Stapf) R.D. Webster and cross-amplification in other Urochloa species
Source: BMC Res Notes. 2016 Mar 10;9:152. doi: 10.1186/s13104-016-1967-9 (PMC4785737; doi:10.1186/s13104-016-1967-9)
Supplement: Supplementary file 1 — 10.1186/s13104-016-1967-9 Transferability of the SSR markers developed for Urochloa decumbens. [file 13104_2016_1967_MOESM1_ESM.docx]

**Additional File 1. Transferability of the SSR markers developed for *Urochloa decumbens***

| **Transferability ^a, b^** |  |  |  |  |
| --- | --- | --- | --- | --- |
| **SSR locus** | ***U. dictyoneura*** | ***U. ruziziensis*** | ***U. brizantha*** | ***U. humidicola*** |
| Dec01 | 1/2 | 1/2 | 1/2 | 0/2 |
| Dec03 | 2/2 | 1/2 | 2/2 | 2/2 |
| Dec05 | 2/2 | 2/2 | 2/2 | 2/2 |
| Dec06 | 2/2 | 2/2 | 2/2 | 2/2 |
| Dec07 | 0/2 | 1/2 | 0/2 | 0/2 |
| Dec09 | 0/2 | 0/2 | 1/2 | 1/2 |
| Dec10 | 2/2 | 2/2 | 2/2 | 2/2 |
| Dec11 | 1/2 | 1/2 | 1/2 | 0/2 |
| Dec12 | 1/2 | 2/2 | 1/2 | 1/2 |
| Dec13 | 2/2 | 2/2 | 2/2 | 2/2 |
| Dec14 | 2/2 | 2/2 | 2/2 | 1/2 |
| Dec17 | 2/2 | 2/2 | 2/2 | 1/2 |
| Dec18 | 2/2 | 2/2 | 2/2 | 2/2 |
| Dec19 | 1/2 | 2/2 | 2/2 | 0/2 |
| Dec20 | 0/2 | 2/2 | 2/2 | 0/2 |
| Dec21 | 0/2 | 2/2 | 2/2 | 0/2 |
| Dec22 | 0/2 | 2/2 | 0/2 | 1/2 |
| Dec24 | 2/2 | 2/2 | 2/2 | 0/2 |
| Dec26 | 0/2 | 2/2 | 2/2 | 0/2 |
| Dec27 | 2/2 | 2/2 | 2/2 | 2/2 |
| Dec28 | 0/2 | 2/2 | 2/2 | 0/2 |
| Dec29 | 0/2 | 2/2 | 2/2 | 0/2 |
| Dec30 | 0/2 | 2/2 | 1/2 | 0/2 |
| Dec31 | 0/2 | 2/2 | 0/2 | 0/2 |
| Dec33 | 0/2 | 2/2 | 0/2 | 0/2 |
| Dec35 | 0/2 | 2/2 | 1/2 | 0/2 |
| Dec36 | 2/2 | 2/2 | 2/2 | 2/2 |
| Dec37 | 0/2 | 2/2 | 1/2 | 0/2 |
| Dec39 | 0/2 | 2/2 | 1/2 | 2/2 |
| Dec42 | 2/2 | 2/2 | 2/2 | 2/2 |
| Dec43 | 2/2 | 2/2 | 2/2 | 2/2 |
| Dec44 | 0/2 | 2/2 | 2/2 | 0/2 |
| Dec45 | 2/2 | 2/2 | 2/2 | 2/2 |
| Dec47 | 2/2 | 2/2 | 2/2 | 2/2 |
| Dec48 | 2/2 | 2/2 | 2/2 | 2/2 |
| Dec49 | 0/2 | 2/2 | 1/2 | 0/2 |
| Dec50 | 0/2 | 2/2 | 1/2 | 0/2 |
| Dec51 | 0/2 | 2/2 | 2/2 | 0/2 |
| Dec52 | 0/2 | 2/2 | 1/2 | 0/2 |
| Dec54 | 0/2 | 2/2 | 2/2 | 0/2 |
| Dec55 | 2/2 | 2/2 | 2/2 | 1/2 |
| Dec56 | 2/2 | 2/2 | 2/2 | 0/2 |
| Dec58 | 0/2 | 2/2 | 2/2 | 0/2 |
| Dec59 | 2/2 | 2/2 | 2/2 | 2/2 |
| Dec60 | 2/2 | 2/2 | 2/2 | 0/2 |
| Dec62 | 2/2 | 1/2 | 0/0 | 0/0 |
| Dec63 | 2/2 | 2/2 | 2/2 | 0/2 |
| Dec65 | 2/2 | 2/2 | 2/2 | 0/2 |
| Dec69 | 2/2 | 2/2 | 2/2 | 2/2 |
| Dec70 | 2/2 | 2/2 | 2/2 | 2/2 |
| Dec71 | 2/2 | 2/2 | 2/2 | 2/2 |
| Dec75 | 2/2 | 2/2 | 2/2 | 2/2 |
| Dec76 | 2/2 | 2/2 | 2/2 | 0/2 |
| Dec77 | 0/2 | 2/2 | 0/2 | 0/2 |
| Dec78 | 2/2 | 2/2 | 2/2 | 2/2 |
| Dec83 | 2/2 | 2/2 | 1/2 | 1/2 |
| Dec84 | 0/2 | 2/2 | 1/2 | 0/2 |
| Dec86 | 1/2 | 2/2 | 2/2 | 2/2 |
| Dec89 | 0/2 | 2/2 | 1/2 | 0/2 |
| Dec90 | 2/2 | 2/2 | 2/2 | 2/2 |
| Dec91 | 1/2 | 1/2 | 2/2 | 2/2 |
| Dec92 | 0/2 | 2/2 | 2/2 | 0/2 |
| Dec93 | 0/2 | 2/2 | 1/2 | 0/2 |
| Dec95 | 0/2 | 2/2 | 1/2 | 0/2 |
| Dec96 | 0/2 | 2/2 | 1/2 | 0/2 |
| Dec97 | 0/2 | 2/2 | 2/2 | 0/2 |
| Dec98 | 0/2 | 2/2 | 2/2 | 0/2 |
| Dec99 | 0/2 | 2/2 | 1/2 | 0/2 |
| Dec101 | 0/2 | 2/2 | 2/2 | 0/2 |
| Dec103 | 1/2 | 2/2 | 2/2 | 0/2 |
| Dec105 | 0/2 | 2/2 | 1/2 | 0/2 |
| Dec106 | 2/2 | 2/2 | 2/2 | 2/2 |
| Dec108 | 0/2 | 2/2 | 0/2 | 0/2 |
| Dec109 | 2/2 | 2/2 | 2/2 | 0/2 |
| Dec110 | 0/2 | 2/2 | 1/2 | 1/2 |
| Dec111 | 0/2 | 2/2 | 2/2 | 0/2 |
| Dec112 | 2/2 | 2/2 | 2/2 | 2/2 |
| Dec113 | 0/2 | 2/2 | 1/2 | 0/2 |
| Dec114 | 2/2 | 2/2 | 2/2 | 0/2 |
| Dec115 | 2/2 | 2/2 | 2/2 | 0/2 |
| Dec116 | 2/2 | 2/2 | 2/2 | 0/2 |
| Dec118 | 2/2 | 2/2 | 2/2 | 2/2 |
| Dec121 | 0/2 | 2/2 | 1/2 | 0/2 |
| Dec122 | 0/2 | 2/2 | 2/2 | 0/2 |
| Dec123 | 2/2 | 2/2 | 2/2 | 0/2 |
| Dec124 | 2/2 | 2/2 | 2/2 | 2/2 |
| Dec125 | 2/2 | 2/2 | 2/2 | 0/2 |
| Dec126 | 1/2 | 2/2 | 1/2 | 0/2 |
| Dec127 | 0/2 | 2/2 | 2/2 | 0/2 |
| Dec131 | 1/2 | 2/2 | 1/2 | 0/2 |
| Dec132 | 2/2 | 2/2 | 2/2 | 0/2 |
| Dec133 | 0/2 | 2/2 | 1/2 | 0/2 |
| Dec134 | 1/2 | 2/2 | 2/2 | 0/2 |

^a^Number of successfully amplified genotypes/Number of tested genotypes.

^b^Nomenclatural classification: *Urochloa decumbens* (Stapf) R.D. Webster, *Urochloa dictyoneura* (Figure & De Not.) Veldkamp, *Urochloa ruziziensis* (R. Germ. & C.M. Evrard) Crins, *Urochloa brizantha* (Hochst. ex A. Rich.) R.D. Webster, *Urochloa humidicola* (Rendle) Morrone & Zuloaga.
